# CAHOST: An Excel Workbook for Facilitating the Johnson-Neyman Technique for Two-Way Interactions in Multiple Regression

**DOI:** 10.3389/fpsyg.2017.01293

**Published:** 2017-07-28

**Authors:** Stephen W. Carden, Nicholas S. Holtzman, Michael J Strube

**Affiliations:** ^1^Department of Mathematical Sciences, Georgia Southern University Statesboro, GA, United States; ^2^Department of Psychology, Georgia Southern University Statesboro, GA, United States; ^3^Department of Psychological and Brain Sciences, Washington University in St. Louis St. Louis, MO, United States

**Keywords:** moderation, johnson-neyman, interactions, probing interactions, multiple regression

## Abstract

When using multiple regression, researchers frequently wish to explore how the relationship between two variables is moderated by another variable; this is termed an interaction. Historically, two approaches have been used to probe interactions: the pick-a-point approach and the Johnson-Neyman (JN) technique. The pick-a-point approach has limitations that can be avoided using the JN technique. Currently, the software available for implementing the JN technique and creating corresponding figures lacks several desirable features–most notably, ease of use and figure quality. To fill this gap in the literature, we offer a free Microsoft Excel 2013 workbook, CAHOST (a concatenation of the first two letters of the authors' last names), that allows the user to seamlessly create publication-ready figures of the results of the JN technique.

## 1. Introduction

When a researcher seeks to quantify the linear effect an explanatory variable, *X*, has on a response variable, *Y*, the size of that effect may depend on a second explanatory variable, *M*. For example, a person's blood alcohol content is influenced by the amount of alcohol that person has ingested, but the size of this influence depends on, among other things, the body mass of that person. In such a situation, the two explanatory variables are said to “interact” in their influence on the response variable. Taking the view that *X* is the primary variable of interest or the “focal predictor,” the other explanatory variable *M* is the “moderator.” Thus, the study of how the explanatory variables interact is often called moderation analysis (Cohen et al., 2003; Hayes, 2013b). One of the tools used in moderation analysis is the Johnson-Neyman (JN) technique.

This article describes CAHOST (a concatenation of the first two letters of the authors' last names), an implementation of the JN technique in a Microsoft Excel 2013 macro-enabled workbook (.xlsm) which produces high-quality publication-ready graphics, requires no programming capabilities, and limits error in data entry (e.g., entering coefficients). The target audience is researchers without programming experience who wish to probe interactions. Version 1.0 of the workbook may be found in the Supplementary Material accompanying this article, and future releases may be found at https://sites.google.com/a/georgiasouthern.edu/stephen-carden/research. The following sections will describe the JN technique, the underlying mathematics, detail how the workbook operates on a sheet-per-sheet basis, present a brief example, and conclude.

## 2. The Johnson-Neyman technique

The simplest procedure for investigating the signficance of an interaction is the “pick-a-point” (Rogosa, 1980), or “simple slopes” (Aiken and West, 1991) method in which a few values of the moderator are chosen to be fixed, and the significance of *X*'s effect is investigated at those points with a hypothesis test or by constructing a confidence interval. Although easy to carry out, the drawbacks of this method include the values chosen for the moderator being essentially arbitrary, and only yielding information for those arbitrary points.

When the moderator *M* is continuous, a more complete approach is the JN technique (Johnson and Neyman, 1936). Rather than testing for significance at fixed values of *M*, the JN technique works backwards and solves for the values of *M* for which the effect of *X* on *Y* becomes or ceases to be significant. A slight generalization (Bauer and Curran, 2005) can express, as a function of *M*, the lower and upper bounds for the confidence bands estimating the effect of *X* on *Y*. A graph of the confidence bands makes it easy to see for which values of the moderator the effect of the focal predictor on the response is significant. Where the “pick-a-point” method can be thought of as a local “spotlight” method, the JN technique can be thought of as a global “floodlight” method (Spiller et al., 2013).

Implementing the JN technique is possible if one has a software programming background (e.g., in R or SAS). Our goal is to make the JN technique more widely accessible to those who do not have any programming experience. Additionally, our solution is streamlined in the sense that there is only a single step between the user inputting the data and the creation of a publication-ready graphic. There do exist a handful of ready-made solutions for implementing JN, but they suffer from one of several drawbacks. First, the highest-quality existing implementations are for software solutions requiring expensive licenses. The best known is the PROCESS add-on (Hayes, 2013b) for SAS and SPSS. There are a few problems with this. First, some researchers do not have access to SAS or SPSS, which are prohibitively expensive. Second, in the SPSS variety, the JN graphical output requires “considerable editing” (Hayes, 2013b, p. 242). To be sure, there are free solutions, but they suffer from at least one of the following drawbacks: the graphics are of bad quality, the process is not streamlined in that it requires the user to complete the linear regression and creation of the JN figure separately, or the user experience is intimidating to those without programming experience. For example, the probemod package (Tan, 2015) for R produces a graphic that would require considerable editing before being suitable for publication, and the linear regression must be completed separately beforehand, so the process is not as streamlined as it could be. The “rockchalk” package (Johnson, 2016) produces a higher-quality graphic, but still requires the linear regression to be completed beforehand. Last but not least, Kristopher Preacher maintains the website www.quantpsy.org (Preacher et al., 2006) that allows one to run the JN technique. However, the user must manually enter the coefficients, coefficient variances, and so forth. As these must come from a software program, such as SPSS, one must toggle between software and website. Thus, it is not as seamless as it could be, and it may be prone to error when users enter their coefficients. Additionally, the site produces figures that are not publication-ready. CAHOST, our workbook for implementing JN, is freely available, familiar to researchers of all levels, automates the linear regression, and produces publication-ready graphics.

## 3. Mathematics

This section derives the mathematical expressions used in the JN technique. See Bauer and Curran (2005) for additional details. The model for a multiplicative interaction effect takes the form

(1) $$\begin{array}{r} Y_{i}=\gamma_{0}+\gamma_{1}X_{i}+\gamma_{2}M_{i}+\gamma_{3}X_{i}M_{i}+\epsilon_{i}, \end{array}$$

where *Y*_*i*_ are the response values, *X*_*i*_ are the focal predictor values, *M*_*i*_ are the moderator values, the γ's are regression weights, and ϵ represents a normally distributed random error term with zero mean. By symmetry one can see that the *X* and *M* variables could be interchanged; thus the labeling of focal predictor and moderator is mathematically arbitrary and relates only to the researcher's questions of interest. Given a set of data, the γ's may be estimated by the method of least squares. These estimates will be denoted by $\hat{\gamma }$'s, resulting in the prediction equation

$$Ŷ={\hat{\gamma }}_{0}+{\hat{\gamma }}_{1}X+{\hat{\gamma }}_{2}M+{\hat{\gamma }}_{3}XM.$$

Given the treatment of *X* as the focal predictor, the prediction equation can be rearranged to

$$Ŷ=\left({\hat{\gamma }}_{0}+{\hat{\gamma }}_{2}M\right)+\left({\hat{\gamma }}_{1}+{\hat{\gamma }}_{3}M\right)X.$$

By setting

(2) $$\begin{array}{r} {\hat{\omega }}_{0}\left(M\right)={\hat{\gamma }}_{0}+{\hat{\gamma }}_{2}M\\ {\hat{\omega }}_{1}\left(M\right)={\hat{\gamma }}_{1}+{\hat{\gamma }}_{3}M \end{array}$$

the prediction equation can be expressed compactly as

$$Ŷ={\hat{\omega }}_{0}\left(M\right)+{\hat{\omega }}_{1}\left(M\right)X.$$

This form reinforces the perspective that *Y* is a linear function of *X*_1_, but with an intercept and slope that depend on the moderator *M*. To perform a *t*-test for significance of ${\hat{\omega }}_{1}$, we need to estimate its standard error. For a fixed value of *M*, elementary properties of variance yield the standard error of ${\hat{\omega }}_{1}$,

$${SE}_{{\hat{\omega }}_{1}\left(M\right)}=\sqrt{\text{Var}\left({\hat{\gamma }}_{1}\right)+2M\text{Cov}\left({\hat{\gamma }}_{1},{\hat{\gamma }}_{3}\right)+M^{2}\text{Var}\left({\hat{\gamma }}_{3}\right)}.$$

Then at any fixed value of *M*, the test statistic

$$t=\frac{{\hat{\omega }}_{1}\left(M\right)}{{SE}_{{\hat{\omega }}_{1}\left(M\right)}}$$

can be calculated and compared against the critical values *t*_α/2_ for confidence level α from a *t* distribution. This is the “pick-a-point” method. Although this procedure can determine whether the slope of *X* is significant at individual values of *M*, more information can be gained by finding the regions of significance by setting the expression equal to the critical value from a *t* distribution and solving for the moderator. Notice that the solutions are only meaningful if they are possible values of the moderator, thus the JN technique requires the moderator to be continuous. Solving for the moderator results in a quadratic equation of the usual form with solutions m^*^, m^**^ obtained from

$$m^{*},m^{**}=\frac{-b\pm \sqrt{b^{2}-4ac}}{2a}$$

where

(3) $$\begin{array}{r} a=t_{\alpha /2}^{2}\text{Var}\left({\hat{\gamma }}_{3}\right)-{\hat{\gamma }}_{3}^{2},\\ b=2t_{\alpha /2}^{2}\text{Cov}\left({\hat{\gamma }}_{1},{\hat{\gamma }}_{3}\right)-2{\hat{\gamma }}_{1}{\hat{\gamma }}_{3},\\ c=t_{\alpha /2}^{2}\text{Var}\left({\hat{\gamma }}_{1}\right)-{\hat{\gamma }}_{1}^{2}. \end{array}$$

By default, CAHOST uses the critical value corresponding to a 5% level of significance, or equivalently, a 95% confidence level. There are six distinct possibilities that may arise when the roots of the quadratic are found. These six possibilities can be organized into three cases of two similar sub-cases.

**Figure 1:**
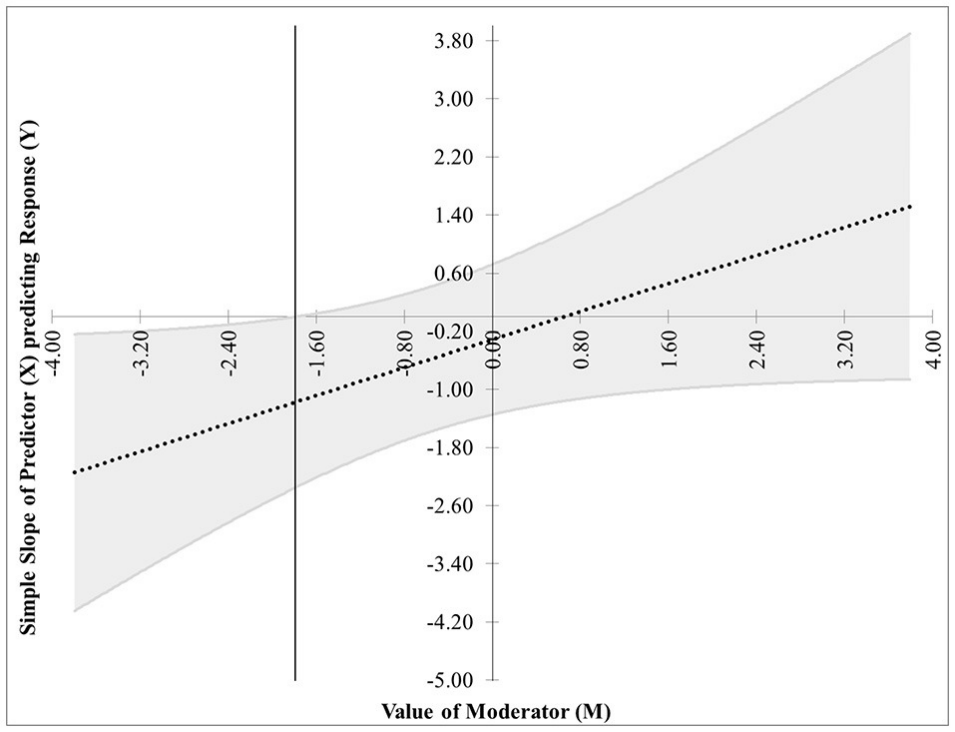
Case 1. At a 95% confidence level, the effect of *X* on *Y* is significant when *M* ≤ m^*^, where m^*^ = −1.79.

**Figure 2:**
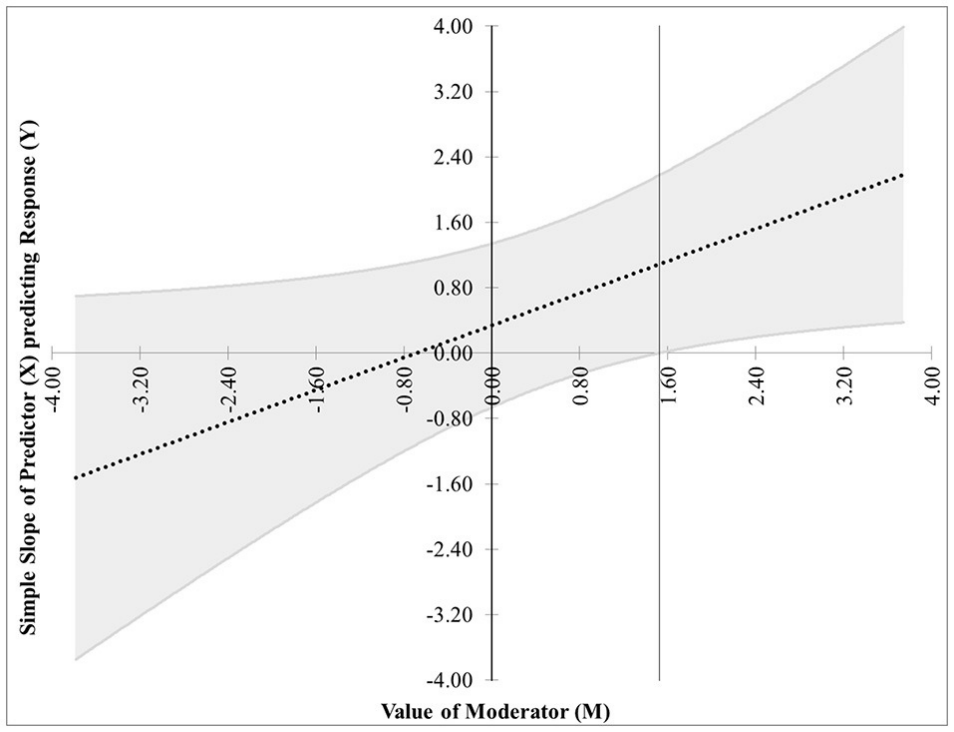
Case 2. At a 95% confidence level, the effect of *X* on *Y* is significant when *M* ≥ m^*^.

**Figure 3:**
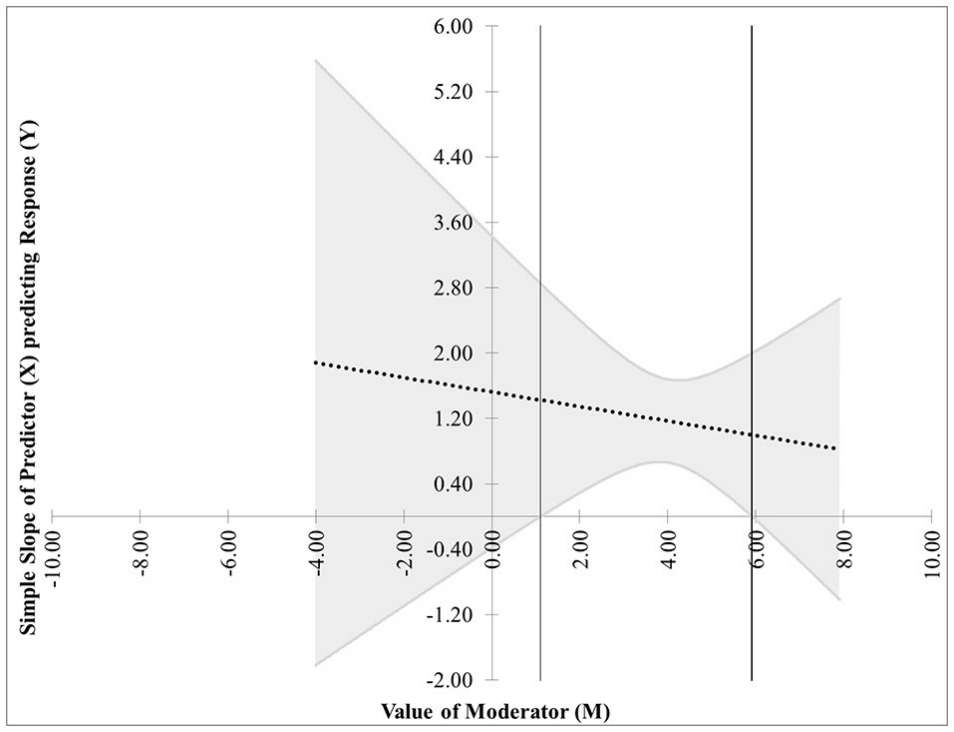
Case 3. At a 95% confidence level, the effect of *X* on *Y* is significant when m^*^ ≤ *M* ≤ m^**^.

**Figure 4:**
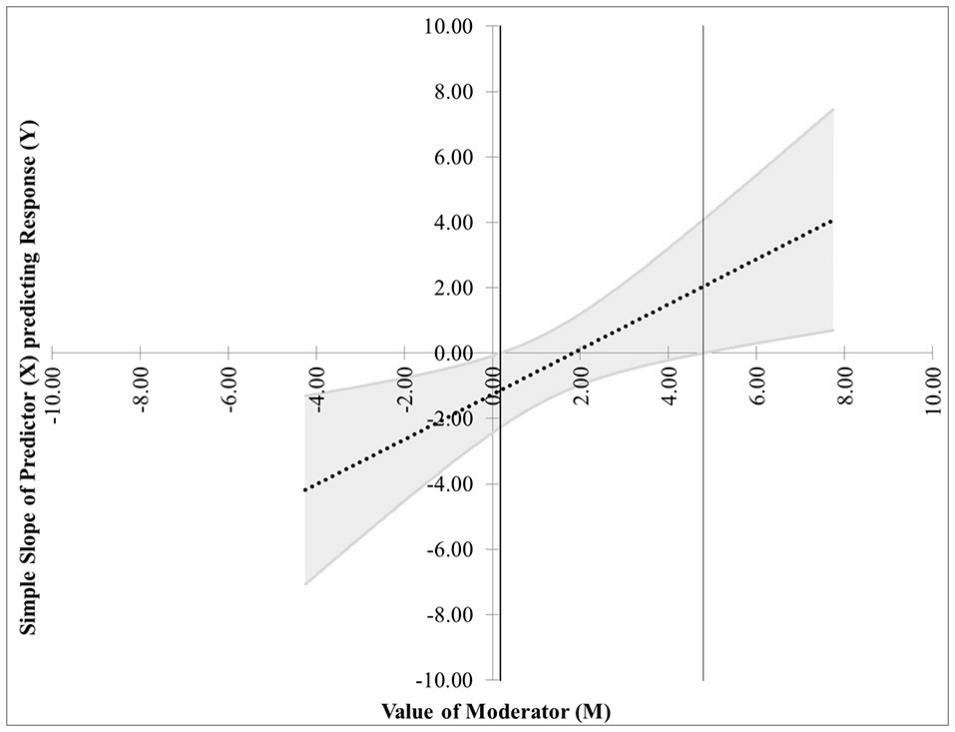
Case 4. At a 95% confidence level, the effect of *X* on *Y* is significant when *M* ≤ m^*^ and *M* ≥ m^**^.

**Figure 5:**
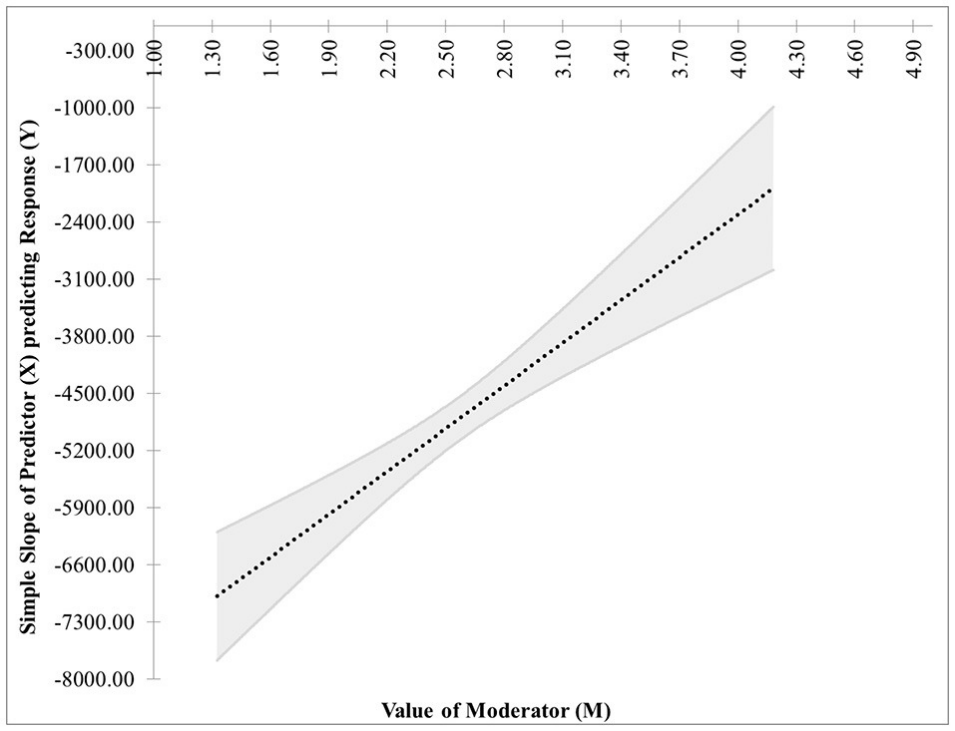
Case 5. At a 95% confidence level, the effect of *X* on *Y* is significant for the entire range of measurements of *M*.

**Figure 6:**
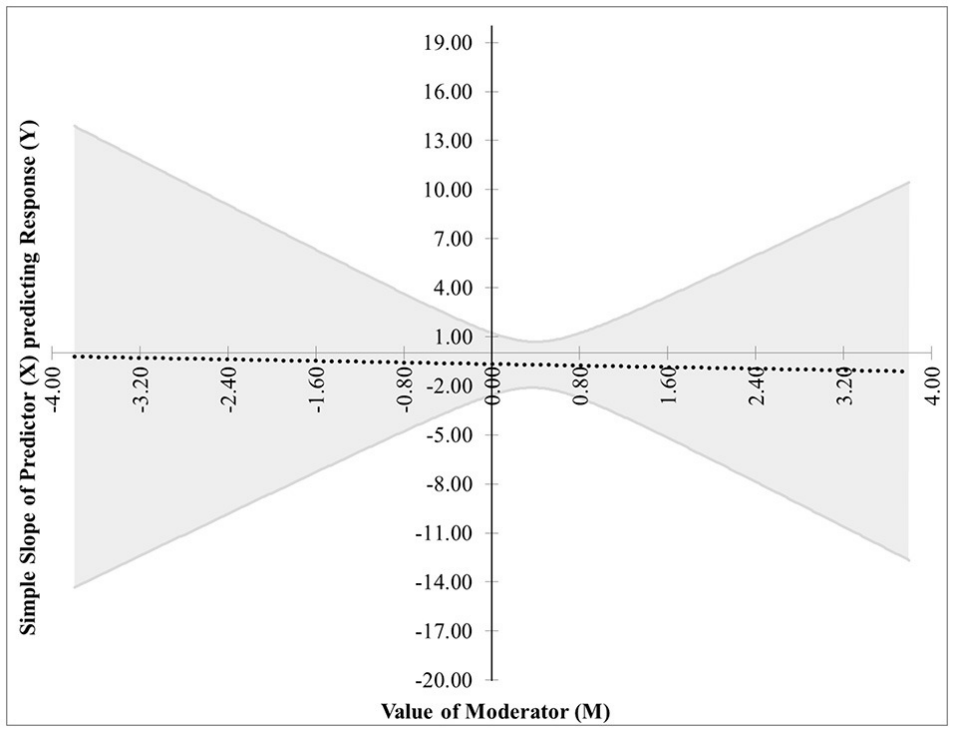
Case 6. At a 95% confidence level, the effect of *X* on *Y* is not significant anywhere in the range of measurements of *M*.

- The first pair of possibilities occurs when two real roots are produced, but only one, call it m^*^, is within the range of measurements of *M*. Then the first possibility occurs when the effect of *X* on *Y* is significant when *M* ≤ m^*^. This is case 1, shown in Figure 1. In this graphic, the horizontal axis represents the values of the moderator within three standard deviations of the mean. On the vertical axis are the corresponding values of the simple slope relating *X* to *Y* as calculated in Equation (2), along with confidence bands and the confidence region shaded a light gray. For any values of the moderator for which the confidence bands contain zero, the effect of *X* on *Y* is not significantly different from zero. Likewise, for any values of the moderator for which the confidence bands do not contain zero, the effect of *X* on *Y* is significantly different from zero. The confidence band crosses zero at m^*^ = −1.79 in Figure 1, with a thin vertical line marking the boundary between regions of significance and non-significance.
  The second possibility occurs when the effect of *X* on *Y* is significant when *M* ≥ m^*^. The graph for this case, Figure 2, is similar to Figure 1 with the difference being that the significance region will be on the right-hand side of m^*^ rather than the left.
- The next pair of possibilities occurs when two real roots m^*^ and m^**^ are produced, and both are within the range of measurements of *M*. One sub-case occurs when the effect of *X* on *Y* is significant when m^*^ ≤ *M* ≤ m^**^ (This is case 3 shown in Figure 3), and the other occurs when the effect of *X* on *Y* is significant when *M* ≤ m^*^ and *M*≥m^**^ (This is case 4 shown in Figure 4).
- The last pair of possibilities occurs when two real roots are produced but neither is within the range of measurements of *M*, or when two complex roots are produced. If the roots are real, then the effect of *X* on *Y* is significant for the entire range of measurements of *M* (This is case 5 shown in Figure 5). If the roots are complex, then the effect of *X* on *Y* is not significant anywhere in the range of measurements of *M* (This is case 6 shown in Figure 6).

Before calculating the roots of the quadratic, consideration should be given to the variance of the error terms. The standard assumption when calculating the matrix containing the estimated variances and covariances of the ${\hat{\gamma }}_{i}$ terms is that the random error terms in Equation (1) are homoskedastic; that is, they have equal variance. If they are actually heteroskedastic and have unequal variances, this introduces inaccuracies in Type I error rates, statistical power, and bounds on confidence intervals. There are several methods for estimating the covariance matrix under heteroskedasticity, and Hayes and Cai (2007) discuss several along with advantages and disadvantages for each. The one we will use is commonly referred to as **HC3** (MacKinnon and White, 1985). Letting **X** denote the data matrix (including a column of ones for the intercept), **HC3** is defined by

(4) $$\text{HC3}={\left({\text{X}}^{⊤}\text{X}\right)}^{-1}{\text{X}}^{⊤}\text{diag }\left(\frac{e_{i}^{2}}{{\left(1-h_{ii}\right)}^{2}}\right)\text{ X}{\left({\text{X}}^{⊤}\text{X}\right)}^{-1}\;\left(4\right)$$

where *e*_*i*_ are the residuals and *h*_*ii*_ are the diagonal entries of the “hat” matrix **X**(**X**^⊤^**X**)^−1^**X**^⊤^. The variances and covariances in Equation (3) are obtained from **HC3**.

## 4. Implementation

CAHOST is written in Microsoft Excel 2013, version 15.0.4911.1000. The workbook consists of 6 sheets. In line with our goal of making the end product as accessible and simple to use as possible, then in the absence of problems with the data, the user needs to interact with the workbook in only two places. Much of the content of these 6 sheets contains information for advanced users and can be skipped by the typical user. The role of each sheet will be described in the following.

The first sheet is titled “1. Read Me” and gives instructions for each subsequent sheet, along with a few bibliographic pointers and acknowledgments. No user input is required.

The second sheet is titled “2. Enter Raw Data.” In cell C4, the user can set the desired significance level, α, to be used. By default, it is set to 5%, corresponding to a 95% confidence level. Below that, in cells C5 through C7, the user may enter names for the variables, which will automatically be incorporated into figure labels in the output sheet. The data for *Y*, *X*, and *M* is placed in columns B, C, and D, respectively. The sheet can accept up to 1,000 observations of each variable. On the right-hand side of this sheet is storage space and example data sets with which the user can experiment and test the capabilities of the workbook. The seven example data sets that come with the workbook are the ones used to create the figures in this article.

The third sheet is titled “3. HC3.” This is where most of the data manipulation and matrix calculations occur. No user input is required on this sheet. The processing of the data will be described on an item-per-item basis below.

- Columns B through E import the data from the previous sheet, determine the number of observations, and check that there are no missing data.
- Columns H through K lay out the observations for which no variable values are missing, add the intercept term, and calculate the interaction term. These columns constitute the data matrix, **X**.
- Columns M through AI carry out the ordinary least squares calculations. Specifically: Columns M through P calculate (**X**^⊤^**X**)^−1^. Columns R and S display regression coefficient estimates. Columns W and X list predicted values and residuals. Columns AA through AC display the SSE, associated degrees of freedom, and MSE summaries. Columns AE through AI display the estimated covariance matrix for the regression coefficient estimates. Note that this covariance matrix is not used in further calculations because **HC3**, the heteroskedasticity-consistent covariance estimator, will be used. It is displayed for the benefit of advanced users.
- Columns AK through BZW carry out the calculations for **HC3**. The “hat” matrix **H** = **X**(**X**^⊤^**X**)^−1^**X**^⊤^ is in columns AK through AMV. The diagonals from this matrix, often termed the *leverage* values, represent the potential influence the corresponding response value has on fitted values. Leverage values equal to one result in an undefined **HC3** matrix; thus, the diagonals are listed below the matrix on row 1010 to aid in error-checking. Columns AMX through ANC calculate the values necessary for the diagonal matrix in the HC3 calculation, and columns ANE through BZQ construct that diagonal matrix. Finally, **HC3** is calculated and displayed in columns BZS through BZV.

The fourth sheet, titled “4. Error Check,” informs the user if the previous sheet encountered any potential problems with the data.

- The first check is to ensure that no observation had missing values for *Y*, *X*, or *M*. The box is green if all values are present, and red if any are missing. This is most likely due to user error in inputting values, or copying and pasting from a source that includes observations with missing values. The user should check the data entered into sheet 2.
- The second check examines the determinant of (**X**^⊤^**X**)^−1^. If this value is a non-zero numerical value, the box is green. If the value is 0 or an error is returned, the box is red. This means the data matrix is not full rank, and usually happens when there is not enough variability in the observations. The user must add enough observations to make the data matrix full rank, or the JN technique cannot be completed.
- The third check looks at the maximum diagonal entry of the hat matrix. If the maximum is less than one, this box is green. If the maximum is one, the box is red, indicating that **HC3** is undefined. This means that some observation has too much leverage, and HC3 cannot be calculated. This is a rare occurance, and if encountered, the user is encouraged to consult with a statistician as to whether the high-leverage observation can be dropped. Otherwise, the user is recommended to use an implementation of JN which does not use HC3 for covariance estimates.
- The fourth check looks at the variance and covariance of the regression coefficients used in the construction of the JN figure. If none of the key values are zero, the box is green. If any of the key values are zero, the box is red. While the figure is still created, no confidence bands appear due to the lack of variability in the estimates. If this occurs, it could be that the data represents a deterministic process with no variability, but it is more likely that the data set is very small and has not yet captured the variability present in the process.

The last two sheets contain the output. The fifth sheet is titled “5. JN Figure.” Columns A through C contain a summary of the matrix and regression calculations from the third sheet. Columns D through P contain calculations used in the creation of the figure. These columns can be ignored by the user. Columns Q and R contain calculations for setting the axes and scale of the figure. Below these calculations is a “Create Figure” button. When the user clicks this button with the figure highlighted, a Visual Basic macro will run and create or update the figure.

Sheet six, “6. Simple Slopes,” constructs a figure using the “simple-slopes” technique mentioned in the introduction. This figure first considers low values of the moderator *M* by setting it to be one standard deviation below the mean. With *M* temporarily fixed, the value of *Y* is predicted at low and high values of the focal predictor variable *X*. The exact values of the focal predictor depend on the values present for *X*. In cells B24 and B25, a calculation determines if the values of *X* are dichotomous. If so, the low and high values are set to be the two distinct values that *X* can take. If *X* is not dichotomous, then it is treated as continuous, and the low and high values are set to be one standard deviation below and above the mean. With *X* on the horizontal axis and *Y* on the vertical axis, the two predicted values of *Y* are plotted and connected with a solid straight line. The slope of this line represents the effect of *X* on *Y* when *M* is below average. This process is repeated with *M* set to one standard deviation above its mean, plotted with a dashed line. The difference in the slopes signifies the moderating effect of *M*. It is not uncommon to see simple slopes graphs that also include a slope for the average value of the moderator, so this sheet has a second graph including the slope at the average of *M*. The user may use whichever of these two graphs they prefer.

## 5. Example

We present an example using data from a workplace gender discrimination study from Garcia et al. (2010). Here we will only apply the JN and simple slopes techniques; a more detailed moderation analysis can be found in Hayes (2013b). The data is available in a compressed zip file from the book's website (Hayes, 2013a). The data consists of 129 women who read a vignette about a female attorney, Catherine, who lost a promotion to a less qualified male. The participants were randomly assigned versions of the story with one of three different endings. In one ending, Catherine did not protest the decision and continued working at the firm. In the other two endings, Catherine protests by requesting that management reconsider their decision. These two endings differ in the manner of protest (making an individual or collective argument), but for the purpose of analysis here, we will group them together. Thus, the story ending is regarded as a binary variable, representing the existence of some form of protest. After reading the story, the participants answer six questions about their perceptions of Catherine, which are combined into a measure of liking. Participants were also given the Modern Sexism Scale instrument, which measures the degree to which the person believes society suffers from sexism. To apply the JN technique, we will treat liking as the response variable, whether Catherine protested as the (binary) focal predictor, and perceived sexism as the moderator.

**Figure 7:**
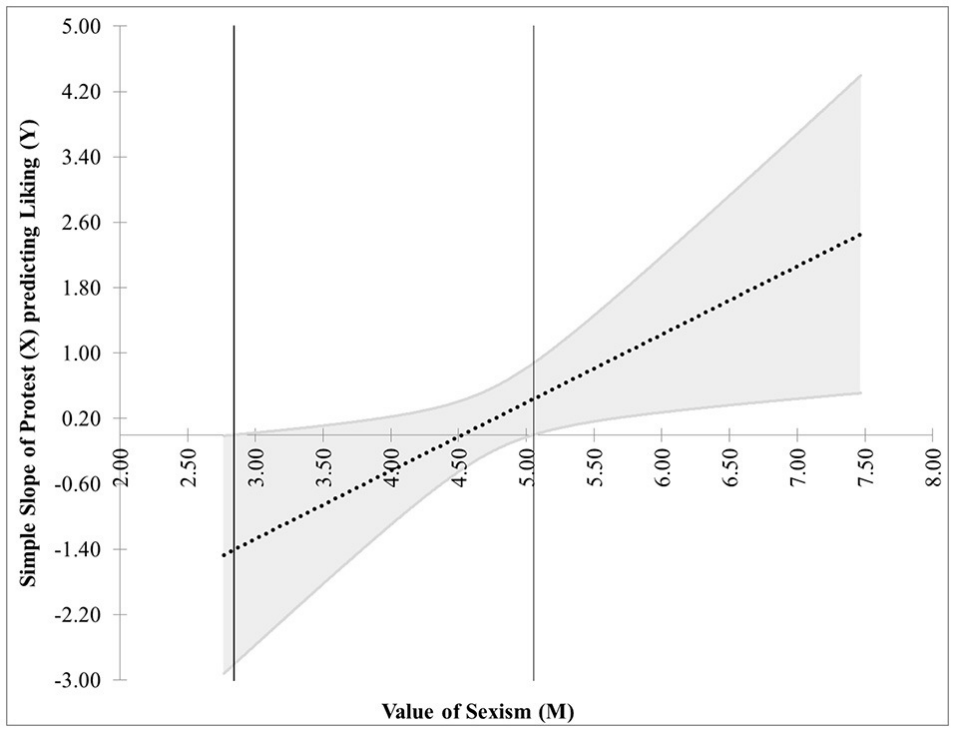
The JN graph for the model relating Liking to Protesting, Sexism, and their interaction. Notice the effect of Protesting on Liking is significant only for high levels of Sexism.

The estimates for the regression coefficients are in Table 1. Figure 7 shows the JN graph for this model. For values of Sexism greater than 5.05, the effect of Protest on Liking is significantly different from zero. Also, notice the figure shows another region of significance for values of Sexism < 2.84. However, while the value 2.84 is within three standard deviations of the mean value of Sexism and thus appears on the graph, the lowest value of Sexism observed in the data is 2.87, and regions of significance close to the extreme values of the measurements should be interpreted with caution. In this case, since the lower region of significance is below the smallest value of the moderator, it is probably best to consider this model to have only one region of significance.

**Table 1:** Regression summary relating liking to protesting, sexism, and their interaction.

**Variable**	**Role**	**Regression coefficient**
Liking	Response	−
−	Intercept	7.7062
Protest	Focal predictor	−3.7727
Sexism	Moderator	−0.4725
Protest^*^Sexism	Interaction	0.8336

**Figure 8:**
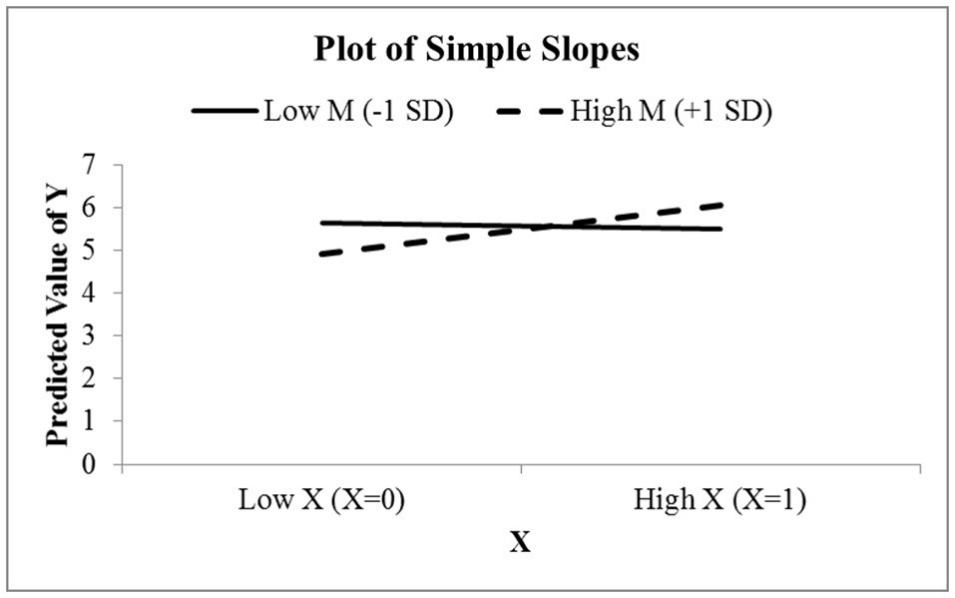
The simple slopes graph for the model relating Liking to Protesting, Sexism, and their interaction. Notice the effect of Protesting on Liking is nearly zero for low levels of Sexism, and positive for high levels of Sexism.

Figure 8 shows the simple slopes graph for this model. Since Protest is a binary condition, CAHOST recognizes *X* as a dichotomous variable, and plots the slope of Protest on liking for low values of Sexism (the solid line), which can be seen is nearly horizontal, agreeing with the JN graph that the effect of Protest on Liking is not significant for low levels of Sexism. The dashed line is the slope of Protest on Liking for high values of Sexism. This slope is positive, in agreement with the JN graph that the effect of Protest on Liking is significant for high levels of Sexism.

## 6. Conclusion

The JN technique is a method for exploring the moderating effect that a continuous variable has on the relationship between a focal predictor and the response variable. This paper has described a workbook intended to make implementing the JN technique as easy as possible for researchers without programming experience or without access to advanced software with significant licensing fees. The use of a heteroskedasticity-consistent covariance estimator, **HC3** allows the workbook to be used in a “one size fits all” manner. Care has been taken to reduce user burden to two actions: inputting the raw data and pressing the “Create Figure” button. For advanced users, intermediate steps in the **HC3** calculations are visible. An error-check sheet alerts the user to any potential problems in carrying out the JN technique. Output includes a summary of the regression analysis, a graphic displaying the regions of the moderator for which the focal predictor's effect on the response is significant, and a graphic displaying the results of a simple-slopes analysis.

Finally, it is necessary to point out some limitations. The workbook is set up to handle up to 1,000 observations. Handling more observations would require significant editing of sheet “3. HC3” or using different software. At the time of this writing, it can only handle two-way interactions, not three-way or higher interactions. Finally, it cannot handle multi-level modeling results. If the user is concerned about these limitations, then we recommend using the www.quantpsy.org website maintained by Kristopher Preacher (Preacher et al., 2006).

## Author contributions

NH and MS conceived the idea for the software. NH and SC developed the software. SC wrote the manuscript. NH and MS provided critical comments on the manuscript. All authors approved the final version of the manuscript for publication.

### Conflict of interest statement

The authors declare that the research was conducted in the absence of any commercial or financial relationships that could be construed as a potential conflict of interest.
